# Modernization, Sexual Risk-Taking, and Gynecological Morbidity among Bolivian Forager-Horticulturalists

**DOI:** 10.1371/journal.pone.0050384

**Published:** 2012-12-06

**Authors:** Jonathan Stieglitz, Aaron D. Blackwell, Raúl Quispe Gutierrez, Edhitt Cortez Linares, Michael Gurven, Hillard Kaplan

**Affiliations:** 1 Department of Anthropology, University of New Mexico, Albuquerque, New Mexico, United States of America; 2 Integrative Anthropological Sciences Unit, University of California Santa Barbara, Santa Barbara, California, United States of America; 3 Proyecto de Salud y Antropología Tsimane, San Borja, Beni, Bolivia; London School of Hygiene and Tropical Medicine, United Kingdom

## Abstract

Sexual risk-taking and reproductive morbidity are common among rapidly modernizing populations with little material wealth, limited schooling, minimal access to modern contraception and healthcare, and gendered inequalities in resource access that limit female autonomy in cohabiting relationships. Few studies have examined how modernization influences sexual risk-taking and reproductive health early in demographic transition. Tsimane are a natural fertility population of Bolivian forager-farmers; they are not urbanized, reside in small-scale villages, and lack public health infrastructure. We test whether modernization is associated with greater sexual risk-taking, report prevalence of gynecological morbidity (GM), and test whether modernization, sexual risk-taking and parity are associated with greater risk of GM. Data were collected from 2002–2010 using interviews, clinical exams, and laboratory analysis of cervical cells. We find opposing effects of modernization on both sexual risk-taking and risk of GM. Residential proximity to town and Spanish fluency are associated with greater likelihood of men’s infidelity, and with number of lifetime sexual partners for men and women. However, for women, literacy is associated with delayed sexual debut after controlling for town proximity. Fifty-five percent of women present at least one clinical indicator of GM (n = 377); 48% present inflammation of cervical cells, and in 11% the inflammation results from sexually transmitted infection (trichomoniasis). Despite having easier access to modern healthcare, women residing near town experience greater likelihood of cervical inflammation and trichomoniasis relative to women in remote villages; women who are fluent in Spanish are also more likely to present trichomoniasis relative to women with moderate or no fluency. However, literate women experience lower likelihood of trichomoniasis. Parity has no effect on risk of GM. Our results suggest a net increase in risk of reproductive morbidity among rapidly modernizing, resource-stressed populations.

## Introduction

A woman’s greatest risk of contracting a sexually transmitted infection (STI) is through unprotected sex with a long-term male partner [1]. Men are more likely than women to bring STI into marriage [2], despite women’s greater susceptibility to STI from intercourse due to anatomical and histological differences in genital organs [3], [4]. While married women are less likely to have multiple concurrent partners compared to their unmarried sexually active peers, married women may have higher rates of reproductive tract infections including STIs [5], [6], [7], . Married women’s risk of STI may be independent of their own sexual behavior [10], suggesting that husbands’ sexual risk-taking mediates the effect of marriage on women’s poor reproductive health. Husbands’ infidelity is associated with increased risk of gynecological morbidity (GM) [8], [11], [12]. GM refers to conditions unrelated to childbirth, such as reproductive tract infections and cervical cell changes [13]. If left untreated, GM can lead to infertility or adverse pregnancy outcomes including fetal loss, infant morbidity, and infant death [14], [15].

Knowledge of factors increasing sexual risk-taking during early stages of modernization is crucial for designing initiatives that improve reproductive health among vulnerable populations. “Modernization” is defined here as a trend toward urban residence and market economy. Rapidly modernizing populations with little material wealth and minimal access to modern contraception and healthcare may experience high levels of risky sexual behavior, such as unprotected sex or sex with an infected partner, and poor reproductive health [16]. These populations have high unmet needs for family planning and limited knowledge of prevention of STIs. Gender inequalities in resource access may facilitate risky sexual behavior and poor reproductive health by increasing women’s likelihood of engaging in transactional sex, and by limiting women’s autonomy in cohabiting relationships. Limited autonomy may lead to sexual, emotional or physical abuse by a partner as well as partner infidelity.

This paper examines effects of modernization on sexual risk-taking for men and women, and prevalence and determinants of GM among Tsimane Amerindians of Bolivia. Bolivia has one of the highest incidence rates of congenital syphilis worldwide [17], and the third highest rate of cervical cancer incidence and mortality in Latin America [18]. Bolivia is one of the poorest countries in the Western hemisphere [19], [20]: it has the second highest percentage of the population that is malnourished (27%) and living below national rural (77%) and urban (51%) poverty lines in Latin America; it has the lowest per-capita gross national income in South America. Bolivia also has the highest total fertility rate (TFR = 3.2 births per woman), and the lowest percentage of reproductive-age married women using modern contraception (34%) in South America. Bolivia is modernizing more rapidly than most countries in the Western hemisphere: while it has the second lowest urban population as a percentage of total (66%) in South America, it has the second highest urban population growth rate in the last 25 years.

As forager-farmers, the Tsimane are not urbanized and exhibit marked village-level variation in town proximity. Tsimane are a natural fertility population (TFR = 9) with little material wealth, no public health infrastructure, and minimal access to modern healthcare and contraception. High rates of infection and inflammation are evident throughout life [21], [22]. Tsimane inhabit small-scale kin-based villages, lack access to Western media, and maintain their indigenous language (unrelated to Spanish) as a first language. Modernization takes several forms: town visits, sale of horticultural and other products, men’s itinerant wage labor with loggers, farmers or merchants, and village schooling. The effect of modernization on sexual risk-taking and risk of GM early in demographic and epidemiological transition remains poorly understood. We examine these relationships in a society with limited variance in socioeconomic status, no consistent use of modern contraceptives, and limited exposure to Western norms, all of which may influence sexual risk-taking and reproductive health.

The paper has three objectives. The first is to test whether sexual risk-taking increases with modernization using three indicators: town proximity, Spanish fluency, and schooling (proxied by literacy). Sexual risk-taking is proxied by likelihood of infidelity and number of lifetime sexual partners for men, and by age at sexual debut and number of lifetime sexual partners for women. The second objective is to report prevalence of GM using clinical and laboratory data among monogamous women. The third objective is to test whether risk of GM increases with modernization, sexual risk-taking and parity.

### 1.1. Modernization and Sexual Risk-taking

In this section, we consider direct and indirect pathways through which modernization may influence the benefit-cost ratio of sexual risk-taking for Tsimane and other populations in the early stages of modernization. Modernization accelerates rates of sexual maturity, increases access to money, increases opportunities for and benefits of transactional sex, inhibits enforcement of social sanctioning of infidelity or multiple concurrent partnerships, and enhances desirability of characteristics signaling greater access to money and modern services. Modernization entails greater access to quality healthcare and schools, although peri-urban subsistence populations with little stored wealth and limited ability to interact with healthcare professionals may not fully realize these benefits.

For Tsimane men, town proximity is associated with greater probability of wage-related village absenteeism per year (controlling for age and dependency load, unpublished data). Town proximity and visitation creates opportunities for transactional sex, including commercial sex. Throughout the developing world, labor migration facilitates men’s participation in extra-marital relationships by mitigating reputational risk of infidelity, inhibiting enforcement of sanctions by wives and other interested parties, and creating desires for companionship to relieve loneliness [23], [24]. Even in developed countries such as the U.S., residence near urban centers is associated with more permissive attitudes toward extra-marital sex [25].

For women, modernization is associated with accelerated rate of sexual maturity, earlier sexual debut, and weaker adherence to norms valuing pre-marital virginity [26], [27], [28]. Resource stress can accelerate women’s sexual debut and increase likelihood of multiple partnerships [29]. Compared to their rural-dwelling peers, urban-dwelling women in the developing world are more likely to report money as a motivator for sex [30]. Even after holding resource availability constant, town proximity may increase women’s sexual risk-taking because cash is more dispensable. Early sexual risk-taking predicts later sexual risk-taking for both sexes: early sexual debut is associated with greater likelihood of concurrent partners [31], and number of lifetime partners [32].

For Tsimane men, Spanish fluency may increase opportunities for sex. In hypothetical scenarios Spanish fluency is rated by Tsimane women as one of the most important traits in a prospective husband [33]. Fluency increases men’s wage opportunities by facilitating sales negotiations with non-Tsimane Bolivians over wood, thatched roof panels and horticultural products, and by helping men develop relationships with employers. Relative to non-fluent men, fluent men may also be more likely to establish social relationships with non-Tsimane women, which may create further opportunities for sex. Fluent men residing in close town proximity may therefore be particularly likely to engage in risky sexual behavior. While fluency may increase men’s sexual risk-taking, fluency also facilitates communication with health professionals in town, eliminating linguistic barriers to improving health.

For women, the relationship between fluency and sexual risk-taking is less clear. Previous studies of the relationship between women’s “acculturation” and sexual risk-taking focus on low-income immigrants in urban areas of developed nations, and lack a consistent definition of acculturation (measures include linguistic proficiency, residence history, and self-identity). Linguistic acculturation is generally associated with greater sexual risk-taking, including number of lifetime partners [34]. Compared to non-fluent Tsimane women, fluent women interact more with fluent men and may therefore have more opportunities to engage in risky sexual behavior such as sex with an infected long-term partner or transactional sex with a short-term partner.

Schooling may increase early sexual opportunities by facilitating encounters with potential mates, and by freeing adolescents from parental supervision. Yet greater schooling is associated with delayed sexual debut and lower rate of pre-marital sex in developed and developing countries [35], [36], although the mechanism is unclear. The effect of schooling on sexual risk-taking in a subsistence economy with no consistent access to modern contraception remains unexplored. Taken together, this review suggests that among peri-urban subsistence populations, modernization may exert opposing effects on sexual risk-taking through town proximity, “linguistic acculturation” and schooling.

### 1.2. Gynecological Morbidity

Correspondence between laboratory analysis of GM (particularly STIs) and self-reported symptoms is low, as is correspondence between laboratory analysis and clinical observations [37], [38]. Many STIs are asymptomatic and may occur with other infections. Symptoms of certain STIs may also appear among uninfected women. Our literature review is largely restricted to studies utilizing laboratory-based data given greater objectivity.

Representative cross-sectional surveys reveal a high prevalence of GM among subsistence-level populations with limited access to modern healthcare. Prevalence of human papillomavirus infection is 64% among sexually active Guarani Indian women [39]. Fifty-nine percent of reproductive-aged women from highland Papua New Guinea present either trichomoniasis (prevalence 47%), chlamydia (26%), syphilis (4%), or gonorrhea (2%) [40]. Forty-seven percent of rural Gambian women present at least one reproductive tract infection, much of which is of bacterial or fungal etiology [38].

Compared to their rural-dwelling counterparts, women residing near urban centers may experience increased likelihood of vaginitis, painful menstruation, syphilis, and genital ulcers in the developing world [41], [42]. Urban wives are more likely than rural wives to report symptoms of GM such as abnormal discharge, even after controlling for parity and education of either spouse [12]. Town proximity may interact with frequency of antibiotic use to influence subsequent risk of GM (if antibiotic-resistant bacteria show higher rates of survival near town).

The association between men’s and women’s sexual risk-taking and GM is well documented. Early sexual debut, number of lifetime partners and partner’s number of lifetime partners are associated with greater risk of trichomoniasis, chlamydia, gonorrhea, human papillomavirus, herpes, pelvic inflammatory disease, and human immunodeficiency virus [8], [9], [40], [43], [44], [45], [46]. Greater schooling may protect against risk of STI and cervical intraepithelial neoplasia [8], [9], [40], [45], [47], but again, the mechanisms remain unclear.

A woman’s age may exert direct and/or indirect effects on likelihood of GM, and lifestyle factors may confound or interact with such effects. Many studies of GM do not utilize representative samples, further obscuring relationships. Younger women might experience greater risk of bacterial STIs such as chlamydia or gonorrhea, although this age-profile may not generalize [40], [46]. Prevalence of herpes simplex virus-2 and syphilis might increase with age [9], but again, the age profiles may not generalize [42]. After controlling for age, higher parity may be associated with increased risk of both cervical intraepithelial neoplasia, a precursor for cervical carcinoma, and genital prolapse [47], [48]. Hypothesized mechanisms include cervical trauma, prolonged immunosuppression, or hormonal effects on cervical epithelium from multiple births. However, others report no effect of parity on likelihood of reproductive tract infections [8], [41].

## Materials and Methods

### 2.1. Ethics Statement

For all protocols, institutional (UNM and UCSB) IRB approval was granted, as was informed consent at three levels: 1) Tsimane government that oversees projects, 2) village leadership, and 3) study participants before and during procedures. Protocols were conducted privately to ensure confidentiality. After explanation of protocols by bilingual Spanish-Tsimane researchers, consent forms were either signed for literate participants, or verbal approval was granted from non-literate participants with a fingerprint.

### 2.2. Study Population

Tsimane (pop. ∼11,000) reside in the rain forests and savannas of central lowland Bolivia (Beni department). At time of study, villages lacked electricity, running water, waste management, and primary healthcare centers. Schools now exist in the majority of villages (>75%). Use of latrines is rare, and individuals frequently sit on the ground or thatched mats placed on the ground. Bathing is done in rivers or lakes. Menstrual fluids are absorbed using clothing designated for that purpose. Men are not circumcised. Intravenous drug use is nonexistent and few women, if any, report a history of non-vaginal sexual contact.

Tsimane lack norms prohibiting pre-marital sex. A couple is considered married when they sleep together in the same house. In the present sample mean±SD age at first marriage for women is 16.7±2.3. Within marriage there is a belief that a husband’s infidelity leads to his children’s sickness and possibly death. Marriages are stable: both sexes age 45+ report an average of 1.3 lifetime spouses. Divorce is most likely in the first year. Modern contraceptive use is rare: in the present sample, 7% of sexually active women age 16+ report ever using contraception (6% received at least one injection of Depo-Provera and 1% used oral contraceptive pills). No women use contraception consistently.

Villages vary widely in their proximity to three towns (in descending order of size): San Borja (pop. ∼25,000), Rurrenabaque (hereafter “Rurre”, ∼14,000), and Yucumo (∼4,000). A major road connects San Borja to Yucumo (44 km southwest), and Yucumo to Rurre (93 km northwest). Mean±SD village distance from San Borja is 42±22 km, from Yucumo 45±20 km, and from Rurre 105±33 km (n = 70 villages). Common reasons for town visits include seeking medical treatment at local hospitals, selling horticultural products, wood or *jatata* (thatched roof panels), and buying market products (e.g., food, clothing, soap). While individual residence patterns may vary over the life course, Tsimane with greater Spanish proficiency and schooling reside closer to town. Spanish is taught in village schools but schooling is not necessary to attain fluency. Many older Tsimane without any schooling speak Spanish based on a history of interaction with non-Tsimane Bolivians.

Sporadic wage opportunities exist for men. Women rarely earn wages and money is rarely saved. Men are often unaccompanied by wives and children during multi-day wage stints and subsequent town visits. While the vast majority of husbands’ wages are used for family purchases (unpublished data), wives frequently complain to husbands about their excessive consumption of store-bought alcohol [49]. In a preliminary survey conducted among men in a village near San Borja in 2010, 59% (20/34) report ever pursuing commercial sex opportunities (9% report 1 instance, 29% 2–5 instances, 3% 6–9 instances, and 18% ≥10 instances).

### 2.3. Data Collection

#### Sexual risk-taking

Data on husband’s infidelity were collected in six villages from 2006–2010 by JS with assistance from a bilingual Spanish-Tsimane male researcher. Tsimane do not have strong taboos against discussing sexual behavior. Retrospective longitudinal interviews were conducted to assess the timing of affairs, including commercial sex, during marriage. This was done by asking with how many women the individual had had affairs, and which children were born when the affair occurred. In about half of marriages, data were obtained independently from husbands and wives in the same marriage, permitting assessment of consistency in reporting male infidelity (sex differences in reporting do not affect results). Individuals who were divorced or widowed were sampled about sexual behavior during current or latest marriages.

During gynecological exams (described below), women were queried about their history of sexual behavior (age at first intercourse and number of lifetime partners). Women also indicated to the best of their knowledge the number of lifetime partners of current husbands.

#### Gynecological morbidity

Data were collected from 2007–2010 during medical exams conducted by the Tsimane Health and Life History Project. From 2007–2009 a mobile medical team visited 25 villages annually, establishing a temporary clinic in each village to provide healthcare and conduct epidemiological surveys. Gynecological exams and Papanicolaou (PAP) tests were conducted among sexually active women, primarily to screen for cervical cancer (we did not systematically screen for most STIs). A female physician and a bilingual female Tsimane assistant invited women to participate regardless of health or marital status. In 2010 screening within villages was discontinued; women age 40+ were transported to a San Borja clinic for screening and to participate in the project’s ongoing studies of aging. The 2010 sample therefore over-represents older women and under-represents younger women (figure S1). The full sample is 568 women, 13% of whom were sampled twice and 2% sampled three times (n = 668 cases). Pregnant and menstruating women were omitted, as were women that were unmarried (8%) or married polygynously (5%).

Prior to visual inspection, a brief gynecological history was elicited. Women were asked whether they experienced recent genital bleeding, discharge, pelvic pain, dyspareunia, or genital itching. Before inserting vaginal speculum, external genitalia were inspected in the dorsal lithotomy position for presence of erythema, edema, and abrasion. After inserting the speculum the vaginal mucosa and cervix were inspected for erythema, edema, discharge, atrophy, and lesions. Presence of vaginitis was recorded, defined as any inflammation (e.g., swelling, redness) of the vaginal canal, with or without discharge. Presence of abnormal discharge was recorded by inspecting the amount, consistency, color, and odor of the discharge. A cervical specimen was taken, placed on a slide and sprayed with fixative. Slides were transported to the *Instituto Nacional de Laboratorios de Salud* (INLASA) in La Paz for analysis. Adequate PAP samples were obtained for 82% of cases and samples lacking adequate cell numbers were omitted. Cervical specimens were evaluated for presence of inflammation. Presence of microorganisms was recorded using three categories: bacterial (generally *Gardnerella vaginalis*), fungal (*Candida albicans*), and trichomonal (*Trichomonas vaginalis*). The Bethesda system was used to code diagnoses, and women were treated by project physicians based on clinical and laboratory findings.

#### Demographics

Age was estimated based on a combination of methods described elsewhere [50]. Parity, residence histories, Spanish fluency, and literacy were assessed during medical exams, demographic interviews, and census updates.

### 2.4. Evaluating and Correcting Sample Bias

Complete gynecological exams (hereafter “GM sample”) were conducted among 28% of women age 18+ that received routine medical check-ups (n = 1624), which in turn represents approximately 75% of the adult female population. Controlling for age, there were no significant differences in fluency or literacy between women who received a gynecological exam and those that received a check-up without a gynecological exam. Additionally, no significant differences emerged between groups in height, weight, body fat, hemoglobin and leukocyte counts, or presence of bacteria or blood in the urine. GM sample participants were significantly more likely than non-participants to be diagnosed by the physician with genital/pelvic problems (OR = 9.30, p<0.001) and STI (OR = 9.12, p<0.001), but not urinary tract infections. Although some diagnoses may have been coded or revised after gynecological exams were conducted, the majority of diagnoses reflect patient complaints or physician assessments made independently of gynecological exams, suggesting that women with genital/pelvic problems were more likely to consent to gynecological exams, or that project physicians were more likely to conduct gynecological exams among these women.

For most of adult life, younger women are less likely than older women to receive gynecological exams regardless of physician diagnosis (figure S2). Age interacts with physician diagnosis: younger women that were not diagnosed were especially unlikely to receive a gynecological exam. To correct for this sample bias, we estimated sample weights by fitting a logistic model of age, physician diagnosis of any genital/pelvic problem or STI, and an age-by-diagnosis interaction term on likelihood of receiving a gynecological exam. Parameter estimates were converted to weights by calculating the inverse of the sampling probability ((1+e^β^)/e^β^) [51]. These sample weights indicate the number of individuals in the population that a person represents based on their age and physician diagnosis. While weights are included in models estimating population prevalences and geographical distributions, the impact of weighting on results is generally modest (see Supplementary Material).

### 2.5. Data Analysis

Likelihood of husband’s infidelity and GM are modeled using the generalized estimating equations (GEE) method, which accounts for correlation of repeated measures on the same individual over time [52]. Regression coefficients are presented as log odds (B) or odds ratios (OR). GEE analyses assume an autoregressive correlation structure since affairs or morbidity may be clumped in time. Where appropriate, p-values are Bonferroni-adjusted to account for the number of tests. Stepwise regression is used to fit some GEE models; starting from a full model variables were removed sequentially in order of highest p-value until all variables were significant at p≤0.10. Stepwise OLS regression is used to examine determinants of sexual risk-taking including husband and wife’s number of partners, and wife’s sexual debut.

For villages classified as near town (section 3.1), mean distance (km) from San Borja, Yucumo, and Rurre = 29, 37, and 89, respectively (n = 32); for remote villages the corresponding distances = 53, 51, and 118, respectively (n = 38).

Continuous geographical models were fit to individual values using generalized additive models (*gam*) in R 2.15.0. Geography was fit with a two dimensional isotropic spline on a sphere, with longitude and latitude as parameters. In models with sample weights, model degrees of freedom were penalized by the mean weight, to avoid over-fitting of splines.

## Results

### 3.1. Is Modernization Associated with Risky Sexual Behavior?

#### Husband’s infidelity

**Figure 1:**
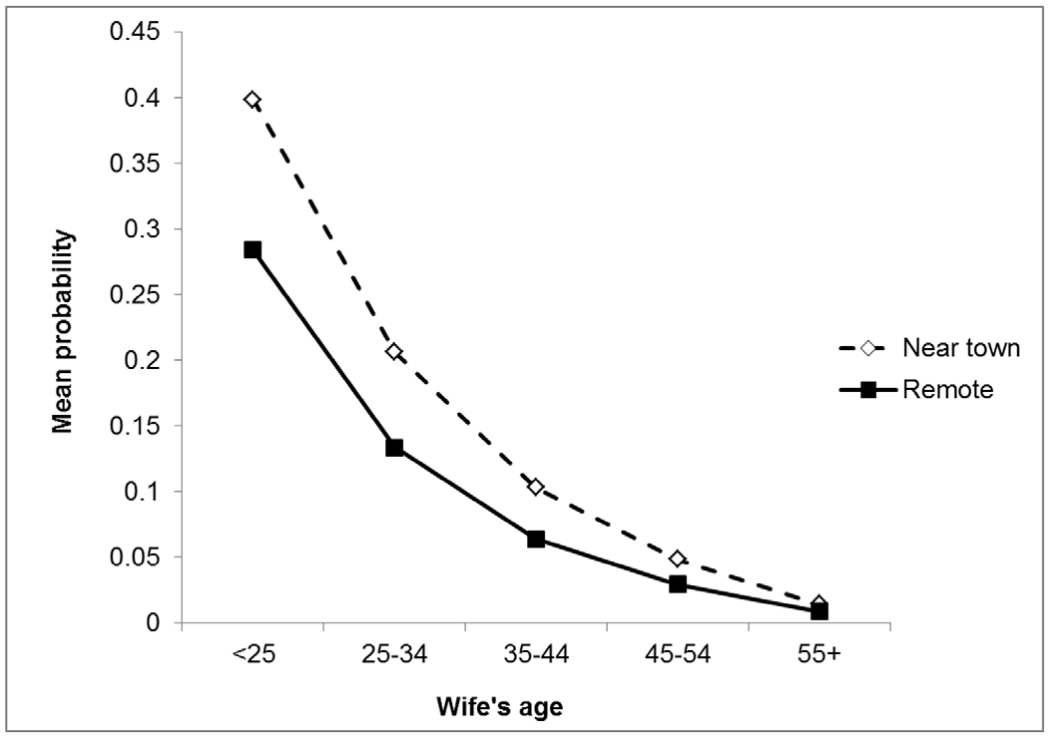
Probability of husband’s infidelity by town proximity and wife’s age. Control variables in table 1 set to sample means (sample min. and max. for wife’s age = 12 and 77, respectively).

Almost 70% of husbands had marital affairs using combined reports of husbands and wives (mean±SD marital risk years = 13±10). Because wives are significantly more likely than husbands to report male infidelity, we control for sex of respondent in multivariate analyses. Close town proximity is associated with 1.7 times greater odds of infidelity (table 1). Infidelity is also more likely when wives are younger (figure 1). Results do not change after controlling for husband’s age, marital duration, or husband’s Spanish fluency or literacy (not shown). Husband’s fluency is marginally associated with rate of infidelity (adjusted OR for fluent vs. no fluency = 2.14, p = 0.1, controlling for covariates in table 1), but literacy has no effect (adjusted OR for literate vs. illiterate = 1.64, p = 0.17).

**Table 1:** GEE analysis of effect of town proximity on likelihood of husband’s infidelity, controlling for wife’s age, presence of children<age 15 and sex of respondent (n = 1,740 marital risk years for 131 husbands).

Parameter	B	SE	p	OR
Intercept	2.471	0.403	<0.001	
Proximity to town = close (ref: remote)	0.524	0.262	0.045	1.69
Wife’s age (years)	−0.082	0.018	<0.001	0.921
Children present (ref: absent)	−0.944	0.195	<0.001	0.389
Respondent sex = female	1.911	0.264	<0.001	6.76

#### Husband’s number of other lifetime partners (wife’s report)

**Figure 2:**
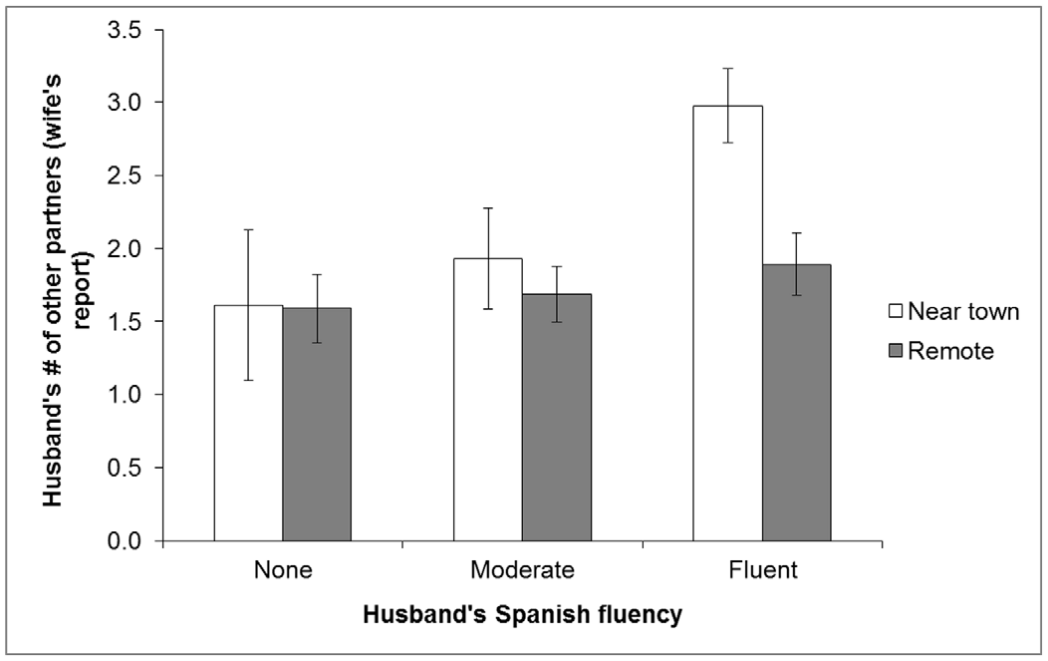
Mean (±SE) number of husband’s other partners (n = 173, controlling for age and age at marriage with a town proximity-by-Spanish fluency interaction term).

Mean±SD number of other lifetime partners is 1.9±1.3 (min. = 0, max. = 15, n = 346). While 94% of wives report ≤3 other partners for husbands, only 1% report no other partners. Marginal mean number of partners for husbands residing near and far from town is 2.5 and 1.8, respectively (p = 0.01, n = 183, controlling for age and age at marriage), and omitting outliers does not affect results. Husband’s Spanish fluency is positively associated with number of partners: marginal mean for fluent men and men with no fluency is 2.3 and 1.7, respectively (p = 0.036, controlling for age, age at marriage, and town proximity). Fluency interacts with town proximity: men residing near town have more partners if fluent (p = 0.046; figure 2). Husband’s literacy has no effect.

#### Wife’s sexual debut

Median age at first intercourse is 15 (mean±SD = 14.7±1.6, n = 448), and 99% of women are sexually active by age 18. Distance from nearest town (km) is negatively correlated with age at first intercourse (r = −0.1, p = 0.05). However, town distance is no longer significant after controlling for wife’s literacy, which is positively associated with age at first intercourse: mean for literate and illiterate women is 15.4 and 14.3, respectively (p<0.001, controlling for town distance). Wife’s Spanish fluency has no effect.

#### Wife’s number of lifetime partners

Mean±SD number of lifetime partners is 1.6±0.9 (min. = 1, max. = 7, n = 456). 58% of wives report one partner. Mean for women residing near and far from town is 1.9 and 1.6, respectively (p = 0.02, n = 239, controlling for age, age at first intercourse, and age at marriage). Wife’s Spanish fluency is positively associated with number of partners: mean for fluent women and women with no fluency is 2.0 and 1.6, respectively (p = 0.05, n = 235, controlling for age, age at first intercourse, age at marriage, and town proximity). Wife’s literacy has no effect.

#### Summary table and geographical distribution of risk factors

**Figure 3:**
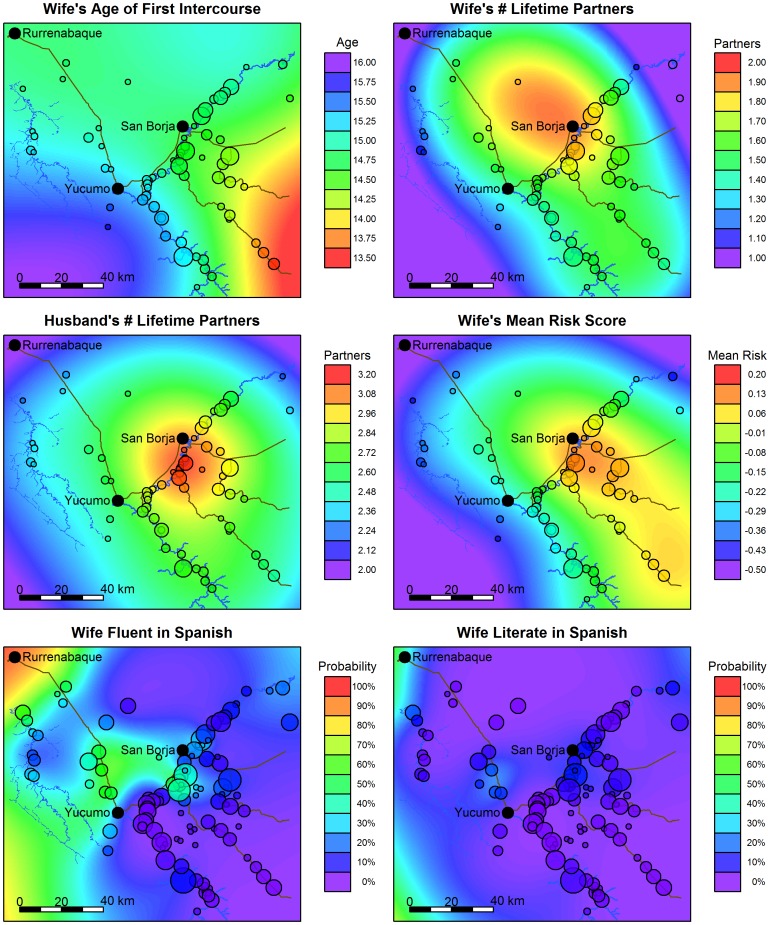
Geographical distribution of risk factors for GM. Distribution of wife’s age at first intercourse and number of lifetime partners for both spouses are based on the GM sample and weighted to adjust for sample bias (see text). To calculate mean risk, wife’s age at first intercourse and number of lifetime partners for both spouses were converted to z-scores and averaged, with age at first intercourse reverse coded. Distribution of Spanish fluency and literacy are based on the sample of women that received medical exams. Circles represent location of study villages, and circle size indicates number of women sampled per village. Estimates are derived from generalized additive models controlling for age. Regions between villages are simulated by the model. Refer to Supplementary Material for geographical distribution without sample weights (figure S3).

Results of models of risky sexual behavior are summarized in table 2. Women’s sexual debut varies little by region (figure 3), although women residing in remote forest villages (southeast of San Borja) may initiate sexual activity earlier than women in other regions. Women and men residing in villages near San Borja have more lifetime partners. Women residing near San Borja and near the Yucumo-Rurre road are also more likely to speak and read Spanish.

**Table 2:** Parameter estimates from OLS regressions of effects of town proximity, Spanish fluency, and literacy on sexual risk-taking.

Dependent variable	Model^b^	Distance toSan Borja(per 10 km)	Distance to Yucumo(per 10 km)	Distance to Rurre(per 10 km)	Husband fluent	Husbandliterate	Wifefluent	Wifeliterate
Husband’s # lifetime partners^a^	Ind	−0.08*	0.01	−0.01	0.64***	0.29	––	––
	STEP	−0.05t			0.60***		––	––
Wife’s age at first intercourse	Ind	0.01	−0.10*	−0.07*	––	––	0.58*	0.98***
	STEP				––	––		0.84**
Wife’s # lifetime partners	Ind	−0.05**	0.01	0.01	––	––	0.29*	0.32t
	STEP	−0.05*			––	––	0.26*	

Models are adjusted for age.

*** p≤0.001;

** p≤0.01;

* p≤0.05; t p≤0.15.

a Wife’s report.

b Each parameter was evaluated independently (Ind), controlling for age. Starting from a full model, parameters were removed in a stepwise fashion until all parameters were significant at p≤0.10 (STEP). To estimate effects on number of lifetime partners (for husband and wife), age at marriage was not controlled as inclusion of this term reduced the effective sample size substantially (given missing data); on a sub-sample where data were available, inclusion of an age at marriage term does not significantly affect results.

### 3.2. Prevalence of GM and Geographical Distribution

Fifty-five percent of women (209/377) present at least one clinical indicator of GM (table 3). Forty-eight percent (201/419) present inflammation from PAP tests. Laboratory diagnosis of persistent inflammation (i.e. inflammation during each visit) reduces prevalence to 18% in a small sample (n = 44 women).

**Table 3:** Prevalence of GM from: A) gynecological exams and B) PAP tests.

	% women presenting at least once
**A) Gynecological exam (n exams)**	
Any GM (421^a^)	55
Vaginitis (476)	51
Abnormal discharge (476)	25
Pelvic pain (433^a^)	18
Dyspareunia (476)	16
Genital itching (476)	12
Genital ulcer (426^a^)	3
**B) PAP test (n tests)**	
Any inflammation (467a, b)	48
* Etiology*	
Bacterial (464)	35
Trichomonal (464)	11
Fungal (464)	2

a Sample size varies due to non-systematic missing data.

b Three women with inflammation of unknown etiology were omitted. The PAP test of one woman revealed inflammation of multiple etiologies (bacterial/trichomonal) and was included.

**Figure 4:**
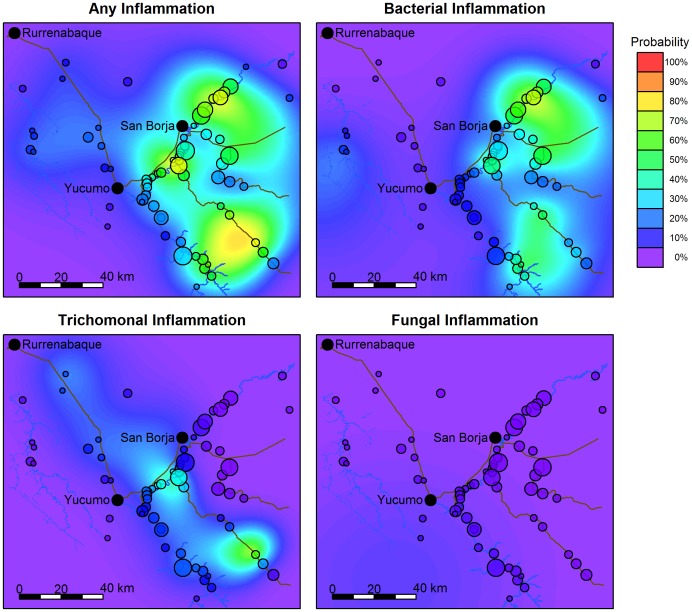
Geographical distribution of GM from PAP tests. Circles represent location of study villages, and circle size indicates relative sample size. Estimates are derived from generalized additive models controlling for age and weighted to adjust for sample bias (see text). Regions between villages are simulated by the model. Refer to Supplementary Material for geographical distribution of gynecological exams (figure S4), and geographical distribution of GM without sample weights (figure S5).

Inflammatory PAP (particularly of bacterial etiology) is more prevalent east of Yucumo (figure 4), in remote upriver Maniqui villages (due south of San Borja) and in downriver Maniqui villages (directly southwest and northeast of San Borja); prevalence is also fairly high in forest villages near San Borja by road, and in remote forest villages. Prevalence is lower in remote villages on the Quiquibey River (south of Rurre). Prevalence of trichomoniasis is greater in villages near the San Borja-Yucumo road, and in one remote forest village with a small sample size. Prevalence of inflammatory PAP of fungal etiology is low, varying little by region.

### 3.3. Effect of Sexual Risk-taking, Town Proximity, and Wife’s Spanish Fluency and Literacy on Likelihood of GM

#### Husband’s number of other lifetime partners

Wives married to men with more lifetime partners experience greater likelihood of abnormal discharge (adjusted OR = 2.24, p = 0.02), pelvic pain (OR = 2.68, p = 0.03), genital itching (OR = 3.38, p = 0.02), and inflammatory PAP of fungal etiology (OR = 9.63, p = 0.05) in stepwise models (table 4). Husband’s number of other lifetime partners is also positively associated with likelihood of vaginitis and inflammatory PAP of bacterial etiology after controlling for age, but not in stepwise models. Contrary to prediction, husbands number of partners is negatively associated with trichomoniasis in stepwise models (OR = 0.27, p = 0.02).

**Table 4:** Odds ratios (ORs) from GEE analyses of effects of sexual risk-taking, town proximity, and wife’s Spanish fluency and literacy on likelihood of GM.

Dependent variable	Model^a^	Husband’s #lifetime partners^b^	Wife’s age at firstintercourse	Wife’s # lifetime partners	Distance toSan Borja(per 10 km)	Distance to Yucumo(per 10 km)	Distance toRurre(per 10 km)	Wife fluent	Wife literate
*Gynecological exam*									
Any GM	Ind	1.48	0.89	1.33*	0.90t	1.03	0.92	1.22	1.43
	STEP				0.90*				
Vaginitis	Ind	1.67t	0.85t	1.49**	0.91t	1.04	0.93	1.42	1.62
	STEP		0.85t	1.42**	0.90t				
Abnormal discharge	Ind	1.88*	0.94	1.26	1.05	0.88	0.94	0.95	0.55
	STEP	2.24*			1.12**	0.81t			
Pelvic pain	Ind	2.73*	1.09	0.91	1.06	1.06	1.02	0.70	0.45
	STEP	2.68*							
Dyspareunia	Ind	1.58	1.08	1.17	1.15*	0.98	0.98	0.36*	0.23*
	STEP			1.59*	1.15t			0.24**	
Genital itching	Ind	2.60*	1.24	1.40t	1.11	1.02	1.04	0.59	0.51
	STEP	3.38*	1.38*	1.51t				0.25t	
Genital ulcer	Ind	0.26	1.06	0.42t	0.88	0.90	0.91	0.45	––c
	STEP				0.05***	0.62*	24.31***		––c
*PAP test*									
Any inflammation	Ind	1.34	1.04	1.16	0.84**	1.11	0.95	1.35	1.31
	STEP				0.82**	1.18t			
Etiology									
Bacterial	Ind	1.74t	1.09	1.27t	0.79***	1.30**	0.92	0.93	1.28
	STEP				0.75***	1.36***			
Trichomonal	Ind	0.51	0.86	0.84	1.11	0.70*	1.09	2.85t	0.96
	STEP	0.27*				0.73*	1.36t	8.93**	0.04***
Fungal	Ind	5.27	1.43	0.30	1.23t	0.68t	1.07	3.03	––c
	STEP	9.63*				0.22**	2.28*		––c

Models are weighted to adjust for sample bias (see text). OR’s are adjusted for age. Refer to table S1 for ORs from unweighted models.

*** p≤0.001;

** p≤0.01;

* p≤0.05; t p≤0.10.

a Each parameter was evaluated independently (Ind), controlling for age and age^2^ if applicable. Starting from a full model, parameters were removed in a stepwise fashion until all parameters were significant at p≤0.10 (STEP).

b Wife’s report; due to skewed distribution and potential for reporting error, husband’s number of partners was coded as: ≤2, >2, or missing. Values represent OR for >2.

vs. ≤2. We cannot test whether husband’s infidelity is associated with greater likelihood of GM due to non-overlapping datasets.

c No literate woman presented genital ulcer or inflammatory PAP of fungal etiology.

#### Wife’s sexual debut and number of partners

Later age at first intercourse is associated with lower likelihood of vaginitis (adjusted OR/year = 0.85, p = 0.06), and a greater likelihood of itching (OR = 1.38, p = 0.04), but not with any laboratory-based indicator of GM (table 4). Wives with more lifetime partners also experience greater likelihood of vaginitis (OR/partner = 1.42, p = 0.02), dyspareunia (OR = 1.59, p = 0.04), and genital itching (OR = 1.51, p = 0.06) in stepwise models. While wife’s number of partners is associated with any GM (OR = 1.33, p = 0.05), genital ulcer (OR = 0.42, p = 0.08), and inflammatory PAP of bacterial etiology (OR = 1.27, p = 0.09), the association is not robust in stepwise models.

Town proximity. Close proximity to San Borja is associated with greater likelihood of any clinical GM and inflammatory PAP (particularly of bacterial etiology) in stepwise models (table 4, figure 4). Close proximity to Yucumo is associated with greater likelihood of trichomoniasis and inflammatory PAP of fungal etiology in stepwise models (table 4, figure 4). After controlling for town proximity, husband’s number of other partners is positively associated with only one laboratory-based indicator of GM (the least prevalent). Wife’s sexual risk-taking is not associated with any laboratory-based indicator of GM after controlling for town proximity.

#### Wife’s Spanish fluency and literacy

Although fluent women are less likely to experience dyspareunia (OR = 0.24, p<0.01) and genital itching (OR = 0.25, p = 0.06) than non-fluent women in stepwise models (table 4), women’s fluency is associated with greater odds of trichomoniasis (OR = 8.93, p<0.01). Literate women experience lower likelihood of trichomoniasis in stepwise models (OR = 0.04, p<0.01); no literate woman presented genital ulcer or inflammatory PAP of fungal etiology.

### 3.4 Effect of Parity on Likelihood of GM

Number of births is not associated with likelihood of any indicator of GM in univariate models, or controlling for age (not shown). To test for a parity effect independent of age (for age effects see figure S6), we regressed number of births on age and modeled residual parity-for-age on likelihood of GM. Higher parity-for-age is not associated with greater likelihood of any indicator of GM.

## Discussion

Modernization exerts opposing effects on sexual risk-taking in a subsistence-level population with traditionally young age at marriage, low divorce rate, limited knowledge of STI prevention, and no reliable access to contraception and healthcare. Rate of men’s infidelity and number of lifetime partners for men and women are greater with increasing town proximity. Men’s solitary itinerant wage labor creates opportunities for transactional sex [49], [53] with reduced risk of social sanctions in small-scale, kin-based villages. Given that men residing near town are more likely than their rural-dwelling peers to pursue wage opportunities, gender inequality in access to modern forms of wealth may increase with town proximity. Limited wage prospects for women increases women’s dependence on men for important market goods and services, thus increasing opportunities for risky sexual behavior in short- or long-term partnerships. As individuals in rapidly developing countries migrate to or near cities in search of employment or schooling, and as rates of sexual maturity and age at first marriage increase with modernization, greater sexual risk-taking is likely to persist [16], [54]. This is particularly likely when wages are sporadic and male-biased, and women cannot support children without assistance from men.

Despite a positive association with number of partners for women, close town proximity is associated with women’s delayed sexual debut. This effect is mediated by women’s literacy (section 3.1). Schooling may decrease sexual risk-taking regardless of whether educational programs address sexual behavior and health [35]. Schooling can enhance communication and planning skills, which also might decrease sexual risk-taking. In addition, schooling can increase interaction with adults discouraging risk-taking, and can reveal economic, health, and other advantages of refraining from risky sexual behavior through abstention or contraceptive use. However, in the present sample, literate women are not more likely to ever use modern contraception compared to women with moderate or no literacy (not shown). In an independent prospective analysis, we find that Tsimane women with more schooling initiate reproduction later than women with less or no schooling (unpublished data). While the mechanisms require further investigation, access to and use of contraceptives per se is unlikely sufficient to explain observed differences in reproductive behavior for women with different levels of schooling.

For Tsimane men, Spanish fluency is associated with marginally greater likelihood of infidelity, and significantly greater number of partners. Interactions with non-Tsimane Bolivians (e.g., employers, medical professionals) may confer economic and health benefits on fluent men, increasing their desirability as short- and long-term partners, particularly near town (figure 2).

For Tsimane women, fluency is not associated with earlier sexual debut but is associated with greater number of lifetime partners. Because fluent women in the present sample do not divorce and remarry at higher rates than women with moderate or no fluency, the increase in number of partners for fluent women may reflect a greater frequency of short-term sexual relationships. Despite early sexual debut at baseline, over half of sampled women report only one lifetime partner (section 3.1). Yet 11% of fluent women report ≥4 partners compared to 3% of non-fluent women, and it is unlikely that this difference results from fluent women’s stronger adherence to sexually permissive norms. In fact, the concept of *marianismo*, the female complement to the male *machismo* gender role prevalent throughout Latin America, emphasizes female passivity and sexual purity. One might therefore expect any change in women’s norms associated with linguistic acculturation to result in fewer, not greater number of partners. One hypothesis is that fluent women have more opportunities and incentives to engage in transactional sex given greater market exposure [29]. In the context of scarce wage opportunities for women, market exposure may give women material aspirations that they can achieve through sexual relations [30].

Prevalence of selected indicators of GM among Tsimane is comparable to that of high risk populations in Bolivia. Cross-sectional analysis reveals a prevalence of trichomoniasis and genital ulcers among Tsimane of 11% and 3%, respectively (table 3). Among female sex workers attending a public health clinic in La Paz, Levine et al. [55] report a comparable prevalence of trichomoniasis (17%) and genital ulcers (6%) prior to implementation of an STI treatment program. Tsimane women exhibit a comparable prevalence of GM despite having a short temporal interval between sexual debut and age at marriage (or no interval for 25%), stable marriages, and limited promiscuity in general (sections 2.2 and 3.1). This is consistent with the idea that husbands’ sexual risk-taking mediates the deleterious effect of marriage on women’s reproductive health [1], [2], [10]. While women’s sexual risk-taking is not associated with any laboratory-based indicator of GM in stepwise models (table 4), men’s sexual risk-taking is associated with greater likelihood of inflammatory PAP of fungal etiology after controlling for town proximity. Contrary to prediction, wives who report more lifetime partners for husbands experience lower likelihood of trichomoniasis. Our results suggest that on top of limited knowledge of STI transmission and limited access to modern contraception and healthcare, Tsimane wives may also be misinformed about their husbands’ sexual histories, particularly outside the bonds of marriage, placing women at further risk of untreated GM.

Despite easier access to modern healthcare near town, Tsimane women residing near town or the road may experience greater risk of GM compared to women living in rural areas (table 4, figure 4). Likelihood of inflammation using clinical (vaginitis) or laboratory data is greatest near San Borja, with a majority of inflammatory cases from PAP tests of bacterial etiology (76% of cases). Bacterial vaginosis (BV) is a common cause of vaginitis and abnormal vaginal discharge among women of reproductive age, yet its pathogenesis remains unclear [56]. BV may or may not be transmitted sexually. Recurrence rates may be high for women previously treated for BV: 23% of Australian women presenting BV at an urban health center experienced recurrence within a month of receiving treatment; 58% within a year [57]. Antibiotic resistance of *G. vaginalis* is being increasingly reported worldwide; among Tsimane higher prevalence of BV near town may result from higher rates of survival of antibiotic-resistant bacteria in the vagina. While in the present sample treatment with metronidazole (an antibiotic commonly used to treat BV) in the previous medical visit (n = 34 cases or 6%) does not increase risk of inflammatory PAP of bacterial etiology in the subsequent visit, residence near town is associated with significantly greater odds of receiving treatment for recent illnesses at hospitals or pharmacies after controlling for age, sex, Spanish fluency, and type of illness (not shown). Tsimane women residing near San Borja thus likely use antibiotics more frequently than their remote-dwelling peers, which might increase prevalence of BV near town.

Spanish fluency may be associated with better access to modern healthcare and better treatment because fluency facilitates communication with medical professionals in town. Yet we find that Tsimane women fluent in Spanish experience greater likelihood of trichomoniasis (table 4). Fluent women are usually paired with fluent men (*r* for husband’s and wife’s Spanish fluency = 0.46, p<0.001, n = 383 couples), and because fluent men are more likely to engage in sexual risk-taking (section 3.1), fluent women may be more likely to engage in sex with an infected partner. Fluent women also report more lifetime partners than non-fluent women (section 3.1), and may be more likely to bring STI into marriage.

The fact that literate Tsimane women experience lower risk of trichomoniasisis is consistent with the finding that literate women delay sexual debut. This is noteworthy given that Tsimane schooling does not include sex education curricula, and that for women schooling per se is not confounded by access to quality healthcare, contraception, or wealth. Understanding causal pathways by which schooling reduces sexual risk-taking, as well as the extent of self-selection, can inform attempts to improve reproductive health in the developing world and should therefore be a priority for researchers.

To conclude, STIs may not be eradicated from the body through the immune response alone, resulting in lifetime infections which may be asymptomatic [58]. There is little empirical evidence that individuals avoid potentially infected mating partners, even when symptoms are present (ibid). Modernization exerts opposing effects on risk of GM, but our results suggest a net increase in risk of reproductive morbidity among rapidly modernizing, resource-stressed populations.

## Supporting Information

Figure S1 **Proportional representation of women receiving vaginal exams and women receiving medical check-ups without vaginal exams (parentheses indicate % receiving vaginal exam).**
(TIF)

Figure S2 **Odds ratio for participation in vaginal exam by age and physician diagnosis.** OR’s derived from generalized estimating equations analysis with an age-by-diagnosis interaction term (n = 2719 check-ups representing 1624 women). The solid green line represents women that were not diagnosed with any genital/pelvic problem or STI; the dashed red line represents women diagnosed with at least one condition. This analysis generated the sample weights included in subsequent analyses (see below).
(TIF)

Figure S3 **Geographical distribution of risk factors for GM without sample weights.**
(TIF)

Figure S4 **Geographical distribution of gynecological exams as a percentage of women receiving medical check-ups.**
(TIF)

Figure S5 **Geographical distribution of GM from PAP tests without sample weights.**
(TIF)

Figure S6 **GEE analysis of effect of age on likelihood of GM.** Panel A refers to gynecological exams and panel B refers to PAP tests; dashed lines represent parameter estimates adjusted for sample weights, and solid lines represent unadjusted estimates.
(TIF)

Table S1 **Odds ratios (ORs) from GEE analyses of effects of sexual risk-taking of both spouses, town proximity, and wife’s Spanish fluency and literacy on likelihood of GM, without sample weights.** OR’s are adjusted for age.
(DOCX)

Text S1 **Supplementary material.**
(DOCX)
