# Supplementary figures and images for: Modernization, Sexual Risk-Taking, and Gynecological Morbidity among Bolivian Forager-Horticulturalists
Source: PLoS One. 2012 Dec 6;7(12):e50384. doi: 10.1371/journal.pone.0050384 (PMC3516519; doi:10.1371/journal.pone.0050384)

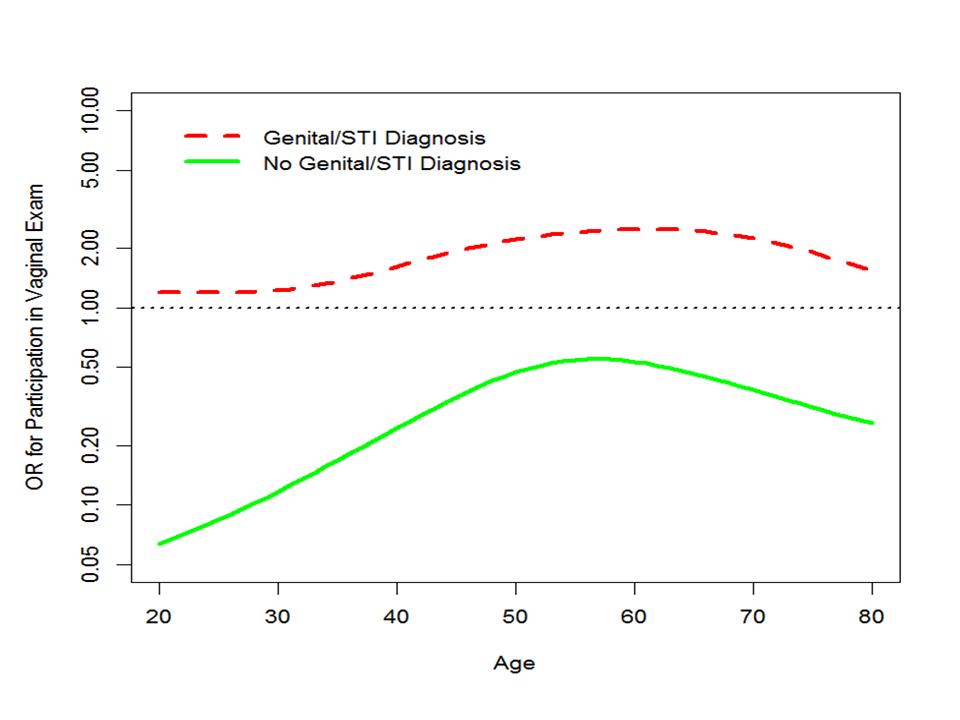

Supplement: Figure S2 — Odds ratio for participation in vaginal exam by age and physician diagnosis. OR’s derived from generalized estimating equations analysis with an age-by-diagnosis interaction term (n = 2719 check-ups representing 1624 women). The solid green line represents women that were not diagnosed with any genital/pelvic problem or STI; the dashed red line represents women diagnosed with at least one condition. This analysis generated the sample weights included in subsequent analyses (see below). (TIF) [file pone.0050384.s002.tif]

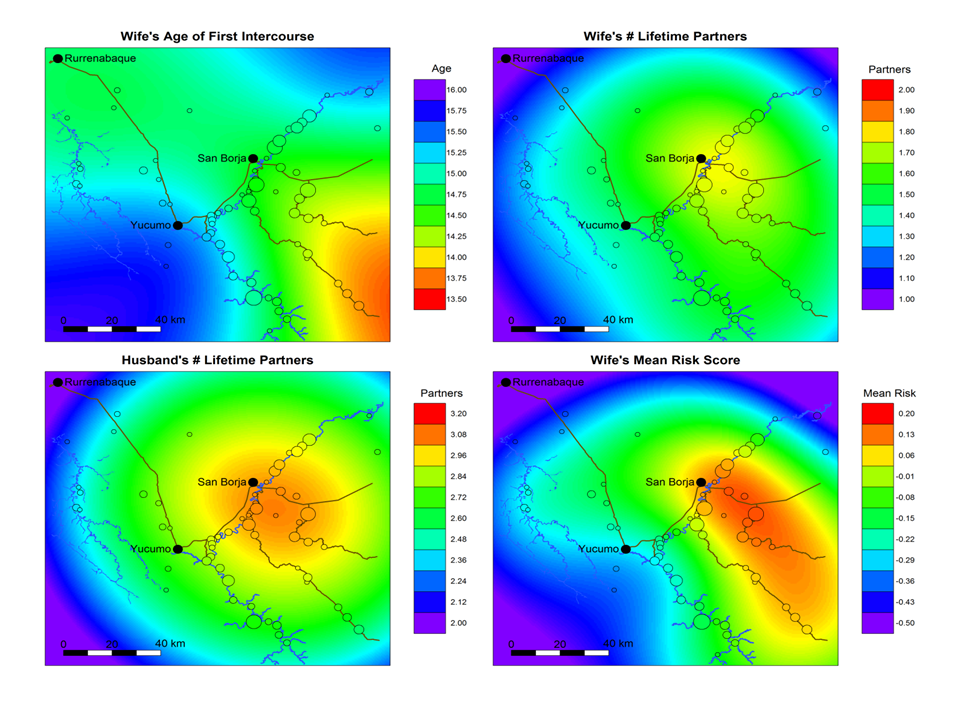

Supplement: Figure S3 — Geographical distribution of risk factors for GM without sample weights. (TIF) [file pone.0050384.s003.tif]

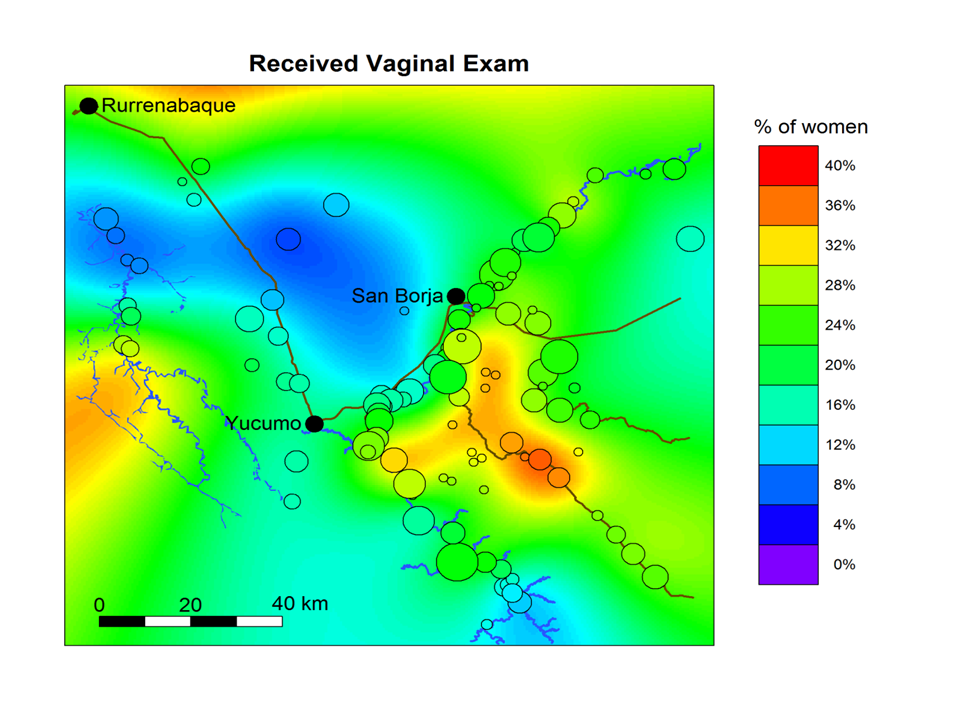

Supplement: Figure S4 — Geographical distribution of gynecological exams as a percentage of women receiving medical check-ups. (TIF) [file pone.0050384.s004.tif]

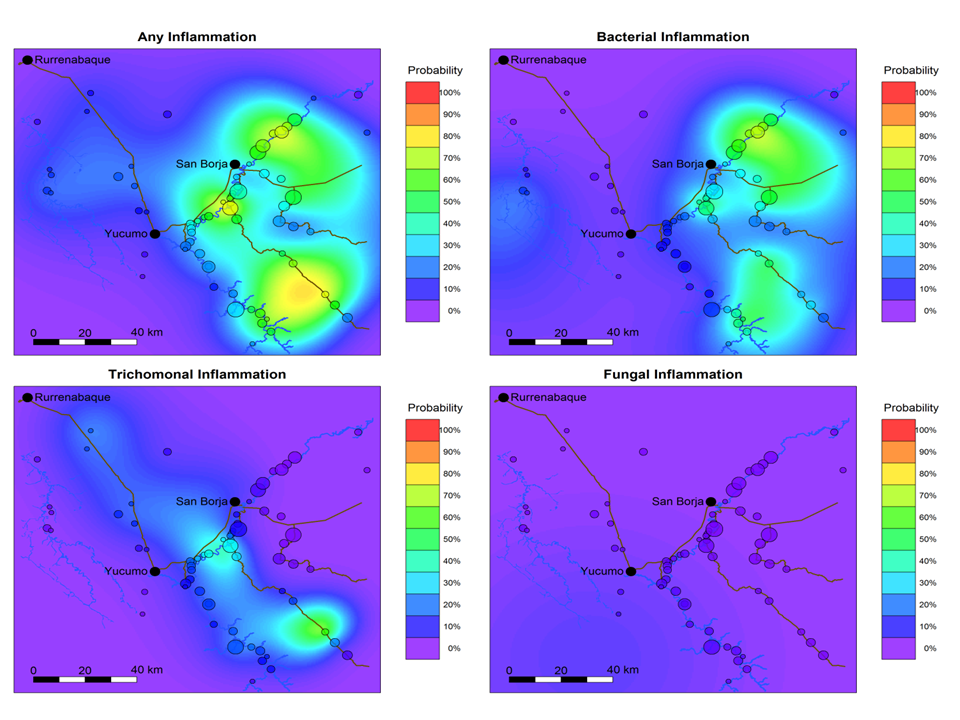

Supplement: Figure S5 — Geographical distribution of GM from PAP tests without sample weights. (TIF) [file pone.0050384.s005.tif]

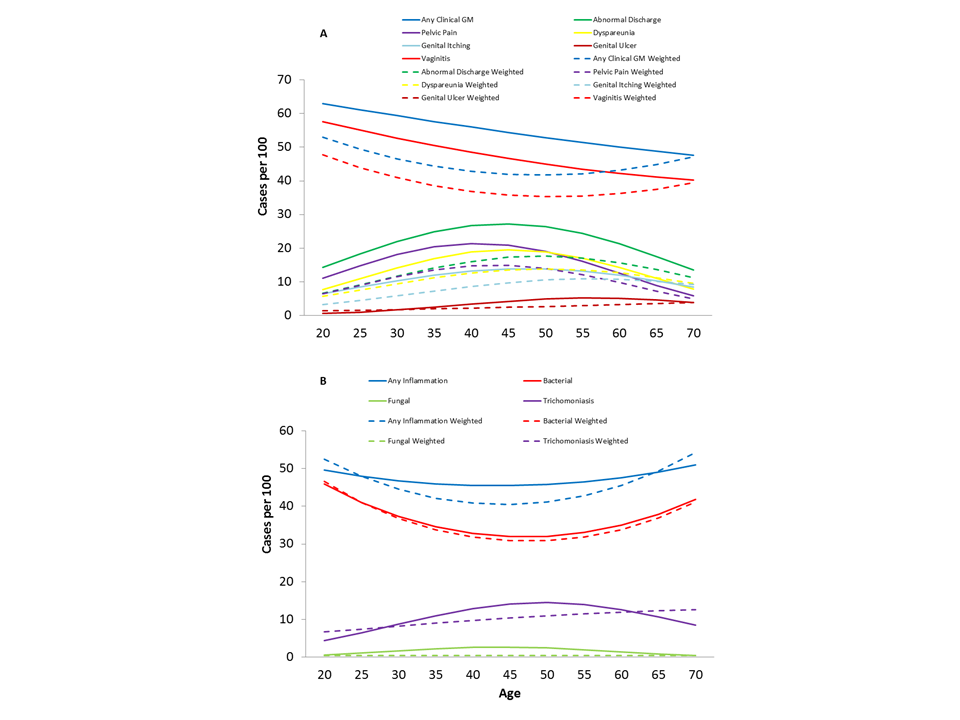

Supplement: Figure S6 — GEE analysis of effect of age on likelihood of GM. Panel A refers to gynecological exams and panel B refers to PAP tests; dashed lines represent parameter estimates adjusted for sample weights, and solid lines represent unadjusted estimates. (TIF) [file pone.0050384.s006.tif]
