# Supplementary material for: Modernization, Sexual Risk-Taking, and Gynecological Morbidity among Bolivian Forager-Horticulturalists
Source: PLoS One. 2012 Dec 6;7(12):e50384. doi: 10.1371/journal.pone.0050384 (PMC3516519; doi:10.1371/journal.pone.0050384)
Supplement: Table S1 — Odds ratios (ORs) from GEE analyses of effects of sexual risk-taking of both spouses, town proximity, and wife’s Spanish fluency and literacy on likelihood of GM, without sample weights. OR’s are adjusted for age. (DOCX) [file pone.0050384.s007.docx]

*Effect of sexual risk-taking, town proximity, and wife’s Spanish fluency and literacy on likelihood of GM*

Table S1. Odds ratios (ORs) from GEE analyses of effects of sexual risk-taking of both spouses, town proximity, and wife’s Spanish fluency and literacy on likelihood of GM, without sample weights. OR’s are adjusted for age.

| **Dependent variable** | **Model^a^** | **Husband’s # lifetime partners^b^** | **Wife’s age at first intercourse** | **Wife’s # lifetime partners** | **Distance to San Borja (per 10 km)** | **Distance to Yucumo**  **(per 10 km)** | **Distance to Rurre**  **(per 10 km)** | **Wife fluent** | **Wife literate** |
| --- | --- | --- | --- | --- | --- | --- | --- | --- | --- |
| *Vaginal exam* |  |  |  |  |  |  |  |  |  |
| Any GM | Ind | 1.33 | 0.87* | 1.32* | 0.91* | 1.02 | 0.93* | 1.00 | 0.82 |
|  | STEP |  | 0.88* | 1.30* | 0.91* |  | 0.91* |  |  |
| Vaginitis | Ind | 1.52t | 0.82** | 1.41** | 0.96 | 1.08 | 0.95 | 0.96 | 0.76 |
|  | STEP |  | 0.85** | 1.44** |  |  |  |  |  |
| Abnormal discharge | Ind | 1.24 | 0.91 | 1.07 | 1.10t | 0.97 | 0.94 | 1.08 | 0.33t |
|  | STEP |  |  |  | 1.19** |  | 0.89* |  | 0.33t |
| Pelvic pain | Ind | 1.97* | 0.98 | 1.33* | 0.95 | 1.05 | 1.00 | 1.08 | 0.84 |
|  | STEP | 1.88t |  |  |  |  |  |  |  |
| Dyspareunia | Ind | 1.37 | 0.97 | 1.20 | 1.11t | 1.01 | 0.94 | 0.84 | 0.61 |
|  | STEP |  |  | 1.38t | 1.13t |  |  |  |  |
| Genital itching | Ind | 1.86 | 0.95 | 1.28t | 1.13t | 1.04 | 0.97 | 1.06 | 1.09 |
|  | STEP | 2.37t |  |  | 1.17* |  |  |  |  |
| Genital ulcer | Ind | 0.53 | 1.02 | 0.59 | 0.89 | 0.89 | 0.85 | 0.71 | -----^c^ |
|  | STEP |  |  |  | 0.09** |  | 11.62** |  | -----^c^ |
| *PAP test* |  |  |  |  |  |  |  |  |  |
| Any inflammation | Ind | 1.32 | 0.98 | 1.06 | 0.89** | 1.12* | 1.03 | 1.07 | 1.29 |
|  | STEP |  |  |  | 0.88** | 1.13t |  |  |  |
| Etiology |  |  |  |  |  |  |  |  |  |
| Bacterial | Ind | 1.72* | 0.94 | 1.17 | 0.86** | 1.37*** | 1.03 | 0.85 | 1.30 |
|  | STEP | 1.58t |  |  | 0.81*** | 1.39*** |  |  |  |
| Trichomonal | Ind | 0.53t | 1.09 | 0.92 | 1.06 | 0.72*** | 1.00 | 1.99 | 1.09 |
|  | STEP | 0.28** |  |  |  | 0.77* |  |  |  |
| Fungal | Ind | 4.02 | 1.32 | 0.25 | 1.17 | 0.59** | 0.98 | 2.20 | -----^c^ |
|  | STEP | 11.25t |  |  |  | 0.15*** | 2.39* |  | -----^c^ |

***p≤0.001; **p≤0.01; *p≤0.05 ; t p≤0.10

^a^Each parameter was evaluated independently (Ind), controlling for age and age^2^ if applicable. Starting from a full model, parameters were removed in a stepwise fashion until all parameters were significant at p≤0.10 (STEP).

^b^Wife’s report; due to skewed distribution and potential for reporting error, husband’s number of partners was coded as: ≤2, >2, or missing. Values represent OR for >2 vs.

≤2. We cannot satisfactorily test whether husband’s infidelity is associated with greater likelihood of GM due to non-overlapping datasets.

^c^No literate woman presented genital ulcer or inflammatory PAP of fungal etiology.
