# Supplementary material for: Modernization, Sexual Risk-Taking, and Gynecological Morbidity among Bolivian Forager-Horticulturalists
Source: PLoS One. 2012 Dec 6;7(12):e50384. doi: 10.1371/journal.pone.0050384 (PMC3516519; doi:10.1371/journal.pone.0050384)
Supplement: Text S1 — Supplementary material. (DOCX) [file pone.0050384.s008.docx]

**SUPPLEMENTARY MATERIAL**

**MODERNIZATION, SEXUAL RISK-TAKING, and GYNECOLOGICAL MORBIDITY among BOLIVIAN FORAGER-HORTICULTURALISTS**

Jonathan Stieglitz^a^ (corresponding author)

Aaron D. Blackwell^b^

Raúl Quispe Gutierrez^c^

Edhitt Cortez Linares^c^

Michael Gurven^b^

Hillard Kaplan^a^

**Affiliations**

^a^Department of Anthropology

University of New Mexico

Albuquerque, NM, USA

^b^Integrative Anthropological Sciences Unit

University of California-Santa Barbara

Santa Barbara, CA, USA

^c^Proyecto de Salud y Antropología Tsimane

San Borja, Beni, BOLIVIA

**METHODS**

*Evaluating sample bias*

Figures S1-S2 show, respectively, the proportional representation of women receiving vaginal exams by age, and the likelihood of participation in exams by age and physician diagnosis.

*Sample weighting to account for bias*

GM sample bias due to the low representation of younger women not diagnosed with any genital/pelvic problem or STI may result in over-estimation of prevalence among younger women. We find a higher prevalence of all laboratory-based indicators of GM among women diagnosed with any genital/pelvic problem or STI compared to women that were not diagnosed (e.g., % of women with trichomoniasis among diagnosed and not diagnosed=12% and 6%, respectively).

Bias may also result in lower variance in GM prevalence across regions given over-representation of individuals with morbid conditions. Below we evaluate the effect of generated sample weights on geographical distribution of risk factors and likelihood of GM, which provides an estimate of the effect of sample bias on results.

**RESULTS**

*Geographical distribution of risk factors for GM*

Figure S3 shows the geographical distribution of risk factors for GM without sample weights applied.

*Geographical distribution of GM*

Coverage for gynecological exams was greatest in the interior forest southeast of San Borja, and lowest near the Yucumo-Rurre road and the Quiquibey River south of Rurre (figure S4).

Comparing geographical distributions generated from models with sample weights (figure 4) and without sample weights (figure S5), weighting generally accentuates differences between low and high prevalence areas. Weighting also results in a modest reduction in prevalences. As with age-patterns (described below), weighting has a greater effect on clinical results from gynecological exams relative to results from PAP tests.

*Effect of age on likelihood of GM*

We regress likelihood of GM on age, both with and without sample weights, using generalized estimating equations (GEE) analyses (figure S6). While sample weighting generally reduces prevalences, particularly among younger women, weighting exerts a relatively modest effect on GM prevalence by age.
